# The Role of Transparency in Digital Contact Tracing During COVID-19: Insights from an Expert Survey

**DOI:** 10.1007/s10728-024-00485-z

**Published:** 2024-06-23

**Authors:** Dennis Krämer, Elisabeth Brachem, Lydia Schneider-Reuter, Isabella D’Angelo, Jochen Vollmann, Joschka Haltaufderheide

**Affiliations:** 1 Faculty of Education and Social Sciences, University of Münster, Münster, Germany; 2 Faculty of Medicine, Ruhr-University Bochum, Bochum, Germany; 3 Faculty of Health Sciences, University Witten-Herdecke, Witten, Germany; 4 Joint Faculty of Health Sciences Brandenburg, University of Potsdam, Potsdam, Germany

**Keywords:** Digital contact tracing, COVID-19, Qualitative research, Transparency, Privacy, Public health

## Abstract

Health technologies such as apps for digital contract tracing [DCT] played a crucial role in containing and combating infections during the COVID-19 pandemic. Their primary function was to prevent the spread of SARS-CoV-2 by consistently generating and disseminating information related to various events such as encounters, vaccinations or infections. While the functionality of DCT has been well researched, the necessity of transparency in the use of DCT and the consent to share sensitive information such as users’ health, vaccination and location status remains unclear. On one hand, DCT enabled the continuous monitoring of various risk factors, including data-based calculations of infection probabilities. On the other hand, digital monitoring of health risks was closely associated with various uncertainties, such as the ambiguous storage of personal data and its potential future misuse, e.g., by tech companies or health authorities. Our contribution aims to retrospectively analyze the COVID-19 pandemic from a post-pandemic perspective and utilize it as a case study for the implementation of new technological measures. We argue that under the condition of voluntary use of DCT, transparency plays a key role in convincing individuals to install health technologies on their mobile devices, keep them activated and consent to the sharing of sensitive data. We support our argument with qualitative data from an expert survey conducted between 2020 and 2021 and analyzed according to the principles of Grounded Theory.

## Introduction

With the outbreak of SARS-CoV-2, societies all over the world faced the challenge of developing new measures to prevent the spread of COVID-19 infections. Against this backdrop, apps for digital contact tracing [DCT] emerged as a global phenomenon [4, 24, 31, 32, 38]. Especially in the early days of the pandemic, it was suggested that DCT could have a crucial impact on fighting the spread of SARS-CoV-2 [9, 35]. Within the first few months of 2020, governments and non-governmental organizations around the world quickly implemented their respective systems. Most countries opted for privacy-preserving and voluntary use models.

With regard to the society-wide implementation of DCT, transparency, understood as the “degree to which information is available to outsiders that enables them to have an informed voice in decisions and/or assess the decisions made by insiders” [10, 43], has been identified as one key factor for the success of a digital pandemic response. In a broad sense, transparency is often associated with *openness* and *honesty* in disclosing information, promoting democratic governance ideals during crises. Its crucial role in gaining, maintaining, and losing trust in a health technology has been explored throughout the pandemic and in various fields, including governance communication, public policy, solidarity and public health information [14, 17, 41, 45].

With regard to DCT, transparency played a key role as a precondition for voluntary uptake [22, 27, 35], allowing users to be sufficiently informed about the function, meaning and scope of a new health technology in a situation of vulnerability to make informed decisions [2]. However, while theoretical modeling of DCT suggested a significant impact on the spread of infections, in practical implementation scenarios, many DCT measures suffered from low uptake rates, mistrust, and suspicions of governmental surveillance, suggesting a lack of transparency and failed measures of clear communication as one of the major barriers [35].

During the pandemic, the reluctance towards DCT and the role of transparency have been mostly researched from an organization-centric perspective or as an object of governmental decision-making, focusing on questions of availability, amount, and timing of data dissemination [17]. While this narrow perspective on pandemic research focusing on transparency mainly addresses information providers, it seems quite natural given the necessity to implement effective strategies during the pandemic. However, it has been suggested that a more comprehensive approach to transparency may be required to fully understand the issues arising during the pandemic [26].

Our contribution aims to analyze the role of transparency in a digital pandemic response from a post-pandemic perspective. We see the pandemic as a dramatic but at the same time as an instructive setting for understanding the deployment of a new health technology aimed at protecting lives in a situation of global vulnerability. In this regard, we believe that even after the peak of the COVID-19 pandemic there are lessons to be learned about how such health technologies need to be developed and how they should collect and secure data to gain users’ trust. Against this backdrop, we understand transparency as the result of an ongoing social, ethical and technical process involving various stakeholders such as governments, researchers and health authorities who, in order to convince individuals to use DCT, must not only develop health technologies to combat the pandemic but also communicate their functions and purposes in a clear and understandable way. These challenges are not only relevant during a pandemic; they generally elucidate the principles guiding the development of healthcare technologies, the design considerations for the user experience or the strategies for effective communication – with the goal of fostering acceptance and trust in these technologies [5, 30].

To this end, we draw on 19 interviews from an expert survey conducted at the height of the pandemic between 2020 and 2021. Based on our findings, we conclude by specifying four aspects that play a key role in securing transparency in a health technology: communication of complex processes in a comprehensible way, protection of private data, inclusion of critical perspectives and user-friendly design.

## Contact Tracing and COVID-19

Contact tracing is a well-established containment strategy in the history of pandemic outbreaks [16]. Scholars such as the French philosopher Michel Foucault have examined the practice of contact tracing in the Western world by drawing parallels to historical examples such as leprosy, smallpox and the plague [11]. Foucault describes how infected individuals with leprosy in the Middle Ages were often excluded from the community and banished to the outskirts of cities and villages. Their exclusion was closely surveilled and they were prohibited from reentering society. In the efforts to combat the plague, people were assigned specific areas within the cities and villages. These locations were divided into different zones which were segregated based on the assumed infectivity of the individuals. During the smallpox epidemic, for the first time, people living within the regions of a national border were treated as a statistical population. Infections, symptoms and disease progressions were considered alongside other factors such as gender, nationality and age to develop comprehensive containment strategies. Foucault categorizes these approaches as cultural expressions of evolving contemporary societal responses to health crises, dividing them into three types of power: *sovereign* (leprosy), *disciplinary* (plague) and *biopower* (smallpox): “… the leper gave rise to rituals of exclusion … the plague gave rise to disciplinary diagrams … [while smallpox gave rise to] a set of mechanisms through which the basic biological features of the human species became the object of a political strategy, of a general strategy of power.” [11] In this genealogical perspective, DCT exemplifies a contemporary approach to how societies deal with pandemics in the early twenty-first century using the capabilities of new technological measures.

**Fig. 1:**
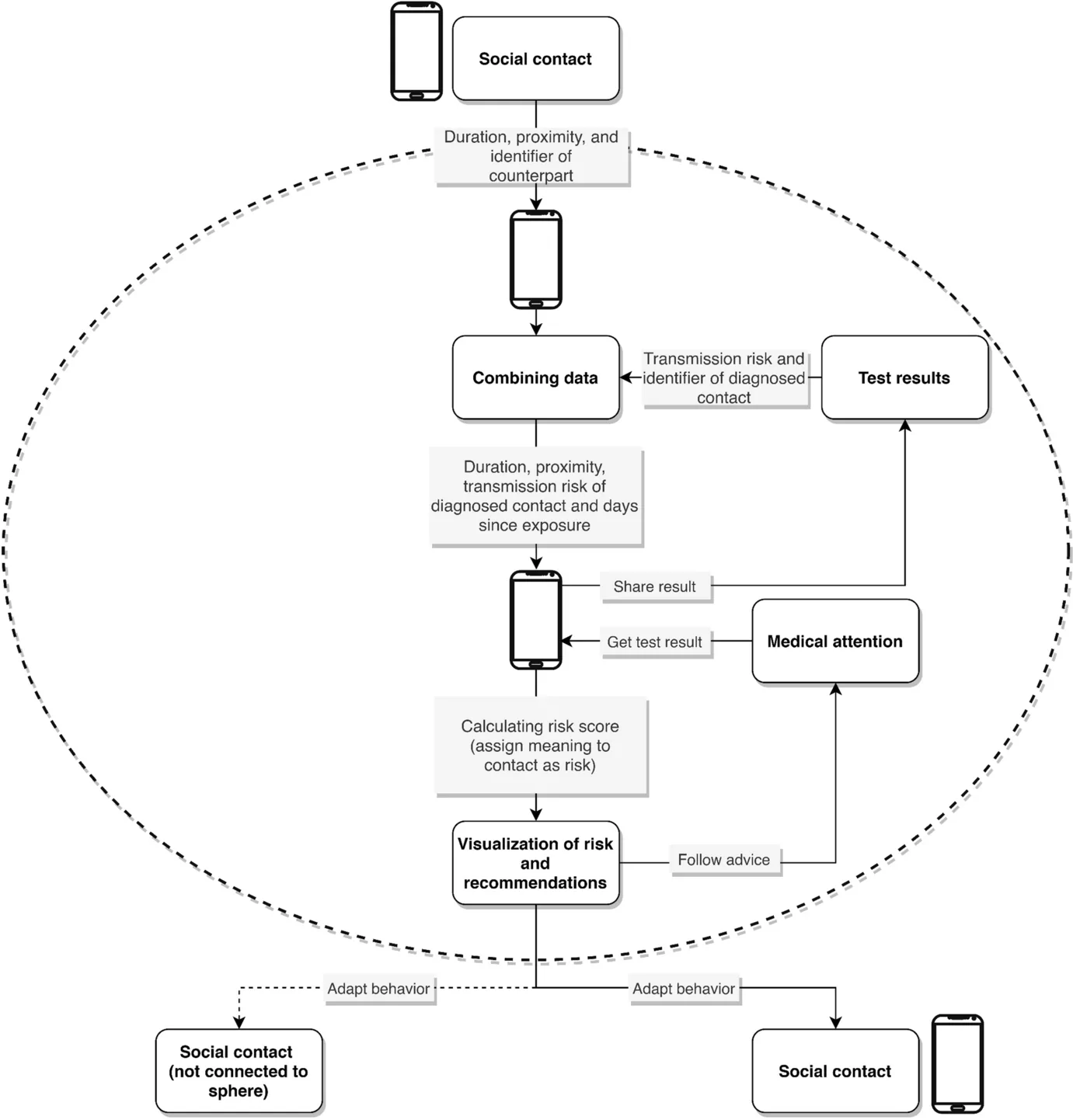
Functionality of digital contact tracing

The primary function of DCT is to map signals from devices as a substitute for physical encounters and to subsequently process these signals to issue warnings or calculate infection probabilities. See Fig. 1 for the functionality of digital contact tracing. However, the operation of this technology and the generation of data depend on the specific socio-cultural context in which it is deployed. As argued by Introna, the development and design of technology can be conceptualized as a process of *closure*, involving a multitude of value-laden decisions where societal and cultural norms influence the arrangement to produce social order [18]. In this sense, as also stated by Latour, the design of technology can be understood as “society made durable” [19, 25]. Certainly, regarding DCT, it has been demonstrated that diverse socio-culturally understandings of privacy and autonomy are integrated into algorithms designed to assess risk exposures [15]. However, due to the technical nature of these systems, such factors and their contributing influences primarily emerge as materialized outcomes, crystallizing in a specific arrangement of technological components that ‘do’ things (calculate risks, process personal health data etc.) – yet, the inner workings of these processes often remain undisclosed to users.

## Methods

### ELISA-Project

The following empirical findings emerged from the interdisciplinary project “The Ethics of Live-Tracking Applications in Connection with SARS-CoV-2” (ELISA), funded by the German Federal Ministry of Education and Research (grant no. 01KI20527), which took place from October 2020 to December 2021. Its primary objective was to gain a deeper understanding of the social, ethical, and technical aspects related to the use of DCT in the pandemic through a qualitative study [14, 15, 23]. To this end, the project focused on the German version of a DCT app, the “Corona-Warn-App”, pursuing various research questions:

- What factors (social, ethical, technical or other) are necessary for individuals to consent to the use of DCT?
- How can they maintain control over their private and health-related data within DCT systems?
- What is the role of transparency in data collection and dissemination, especially in the exceptional situation of pandemic vulnerability?

To achieve its objective, the project was divided into an empirical and an ethical subproject. The goal of the empirical subproject was to create a qualitative database focusing on the identification of critical issues related to the implementation of DCT. The ethical subproject focused on capturing the qualitative findings and subjecting them to an ethical analysis.

## Research Participants

To achieve the goal of gaining a deeper understanding of social, ethical and technical aspects of DCT, and given the exceptional circumstances of the pandemic, the ELISA-team decided to conduct an expert survey. The methodological decision was based on the uncharted scientific landscape on DCT and COVID-19 in the early stages of 2020, as well as the goal of bringing together a broad range of expertise to generate new ideas and solutions by combining multidisciplinary perspectives. In this context, expert surveys can provide access to a knowledge that often remains unknown in public domains due to its specificity and sensitivity [6, 40].

At the same time, they come with various challenges. Among other things, a lack of heterogeneity among the included experts and disciplines can lead to a one-sided representation of the topic under investigation. Additionally, validating discipline-specific statements can be challenging as they often presuppose complex expert knowledge. In light of these challenges, we attempted to address them by striving for a diverse composition of expertise and disciplines. See Table 1 for the assignment of the interviews and academic backgrounds of the interviewees.

**Table 1:** Sample

Interview no.	Gender	Field of expertise
*1*	Male	Journalism
*2*	Male	Ethics
*3*	Male	Ethics
*4*	Male	Computer Science
*5*	Male	Ethics
*6*	Male	Legal Studies
*7*	Female	Social Science
*8*	Female	Medicine
*9*	Female	Social Science
*10*	Female	Medicine
*11*	Male	Legal Studies
*12*	Male	Computer Science
*13*	Female	Medicine
*14*	Female	Ethics
*15*	Female	Medicine
*16*	Male	Computer Science
*17*	Male	Computer Science
*18*	Male	Social Science
*19*	Female	Ethics

Therefore, we needed to identify competent experts who could provide scientifically sound explanations of various aspects of DCT, including their technical functions, legal considerations regarding data processing, storage and privacy, as well as public health issues such as the accuracy of risk calculation algorithms. Experts were defined as scientists from different disciplines who had already worked on DCT or related topics such as health technology or e-health. To achieve this goal, we conducted internal research at the beginning of the project to identify experts and ensure the formation of a multidisciplinary group. Additionally, the project was presented at several conferences and institutions, including medical associations. Potential participants were contacted by email and provided with a brief description of the project. Of the 33 potential interviews contacted, 19 agreed to participate in the ELISA study. 17 of them were affiliated with a German institution, 1 with a Dutch institution and 1 with a British institution. In total, 8 participants were female and 11 were male.

In the following empirical section, the qualitative quotes are presented in an abbreviated form for readability. Therefore, we use a combination of the interview number (1–19), gender (m/f) and field of expertise (Journalism = Journ, Ethics = Eth, Computer Science = Comp, Legal Studies = Leg, Social Science = Soc, Medicine = Med). For example, an interview quote is declared as follows: (7,f,Soc), which means that it is a quote from the seventh interview, conducted with a female interviewee who had expertise in social science.

### Study Design

The ELISA-team consisted of 2 social scientists and 3 ethicists who were supported by a law professor and a student assistant. Both subprojects worked closely together so that the various tasks were addressed within a team of 7 researchers. These tasks included literature reviews, questionnaire development, data analysis, organization of workshops and data sessions as well as collaborative work on publications. One challenge was the limited research available on DCT in the early stages of the COVID-19 pandemic. As the project commenced shortly after the outbreak of SARS-CoV-2, some research findings were only accessible as preprints. To address this, a four-step research process was implemented.

**Fig. 2:**
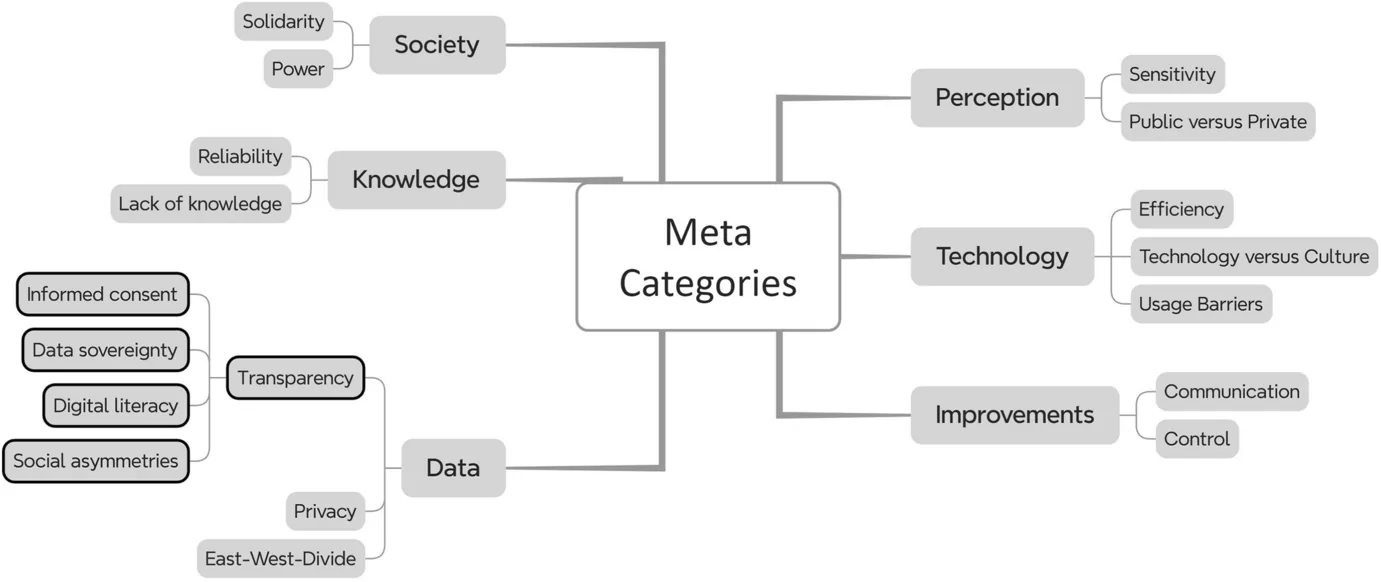
Empirical categories

- *Step 1*: Initially, we created a database to gain an overview of existing publications on DCT. Research questions and identified gaps were documented in a data base and discussed in regular team meetings. Based on this, we developed an initial categorization system, which we iteratively refined as new publications emerged. To empirically address the identified research gaps, we designed a semi-structured questionnaire. In a preliminary step, we conducted a validation testing phase to identify weaknesses and missing connections. For this purpose, we presented the questionnaire during an online workshop attended by 15 researchers including representatives from the Robert Koch Institute (RKI), who were also investigating the role of DCT in the pandemic. The discussions during this workshop were documented and informed the final design of the questionnaire [24].
- *Step 2*: The 19 interviews were conducted between November 2020 and April 2021 by 1 team member from the empirical subproject who was responsible for data collection and transcription. Due to social distancing measures, all interviews were conducted through Zoom. A total of 16 interviews were conducted in German, and 3 in English. For the English interviews, the questionnaire was translated and reviewed by a native speaker. The interview duration varied from 21 to 81 min with an average length of 52 min.
- *Step 3*: The interviews were recorded, transcribed and reflected upon in regular data sessions among the project members. The aim of these sessions was to collaboratively analyze the transcripts, develop consistent interpretations and address research gaps which were identified at the beginning of the project. Preliminary findings were presented and discussed during an online meeting organized by the BMBF in January 2021, as well as within the framework of an organized workshop in September 2021 and an international conference in March 2022 organized by the ELISA staff [44]. These events focused on topics such as the technical implementation of solidarity and social issues related to user participation, awareness or informed consent.
- *Step 4*: The ongoing data analysis is based on an approach of inductive theory generation, following the principles of Grounded Theory [12]. Starting from the perspective that complex explanations about the social reality are grounded in empirical data and need to be worked out, we employed a systematic, iterative approach. Initially, systematic data collection was conducted based on specified criteria, with a focus on the phenomenon under study. The second step involved reading the transcribed interviews and dividing the identified challenges, focal points and issues into smaller segments (open coding). These segments were linked together to identify patterns in the data and establish deeper connections (axial coding). Regular team meetings and data sessions were used for discussions, integrating, deleting, renaming or elaborating on the codes. Coding was managed as an ongoing process, which is currently reflected in 6 meta-categories and 14 sub-categories.^1^ See Fig. 2 for the empirical categories. In the following empirical results section, we focus on the sub-category “Transparency”.

**Fig. 3:**
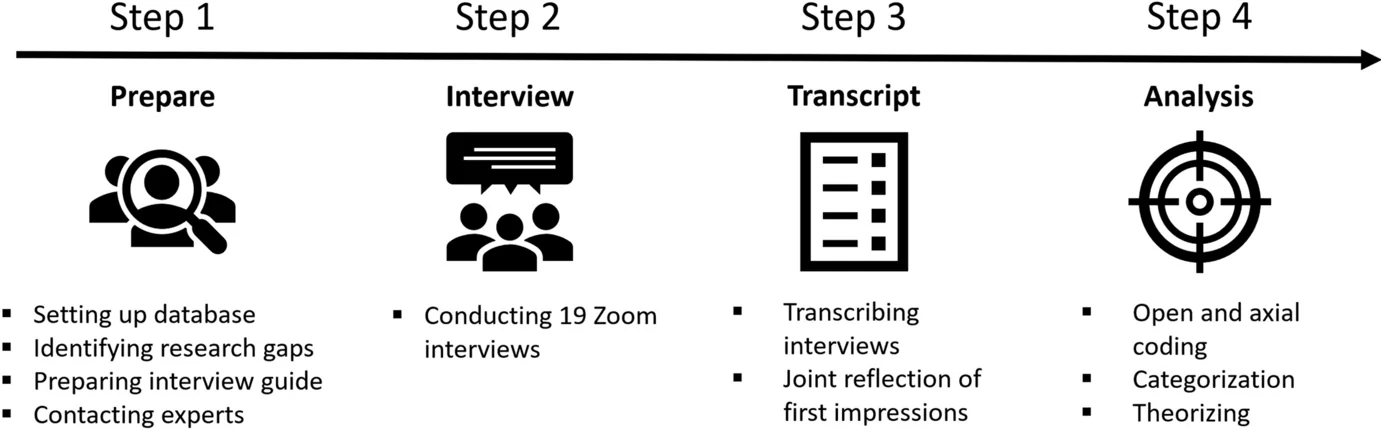
ELISA research process

See Fig. 3 for the research process. Already in the early phase of the project, it became apparent that transparency was not only considered a technical necessity but also played a key role in many other areas in the pandemic situation, such as the calculation of mortality rates, the health policy directive of quarantine regulations or sensitive issues like triage [20]. Our data indicated that transparency was perceived as a complex fundamental condition for handling information which could not be confined to technical questions alone. During data analysis, it became evident that the subcategory “transparency”, according to axial coding based on Grounded Theory, was associated with other categories such as “perception” and “improvements”. As we will demonstrate below, the absence or presence of transparency is thus not only a technical fact but also a social and ethical phenomenon that affects how people perceive a technology, whether they integrate it into their daily lives or reject it [29, 33].

## Empirical Results: Transparency

The outbreak of SARS-CoV-2 has placed many societies in an unprecedented state of vulnerability. The exceptional situation also led to the innovation of technological measures to combat the pandemic. In our study, this area is described as a “completely new field” (4,m,Comp) in an unknown scenario where societies could not “rely on established views” (4,m,Comp) and instead focused on forms of “rigorous contact tracing” (10,f,Med) to render the unpredictability of infections predictable through technologies. In this context, the capabilities of DCT are identified in various social and technical scenarios:

- Reducing the burden on the healthcare system. “In my view, the capability lies firstly in making work easier for health authorities, of course. Every task that an algorithm or app can perform does not necessarily have to be carried out by a person.” (6,m,Leg)
- Expansion of social “responsibility for all individuals” (4,m,Comp). “Yes, [by] basically taking responsibility for all the people you've met in the last few weeks, and these are usually people you like, at least for the most part.” (14,f,Eth)
- Accelerating the exchange of information. That “the test result is then transmitted to the contacts as quickly as possible. This is particularly important if there are very, very many infections. Because then manual contact tracing usually can‘t keep up.“ (10,f,Med)
- Automation through DCT that functions as an interface for reciprocal notifications from the background. “So, of course, it’s very, very helpful … if this chain of infection can be tracked and interrupted, if it runs automatically and without human interaction.” (4,m,Comp)
- Scalability, which enables the expansion of the response to the pandemic by accommodating millions of users. “Because the app is … virtually infinitely scalable. The server doesn’t care whether it sends out ten thousand or a hundred thousand or a million notifications.” (10,f,Med)

In order to fully exploit the capabilities of DCT, our study recognizes a challenge in ensuring the participation of citizens under the condition of voluntary use. In this context, transparency is considered a “protective function” (4,m,Comp) and a “basis of trust” (2,m,Eth) which is not automatically guaranteed but must first be established in order to fulfill the “protective function” (4,m,Comp) of DCT. Following the notes of our experts, this can be achieved “for instance, through comprehensible communication of data protection notices or terms of use” (1,m,Journ), “anonymization” (13,f,Med) or “disclosure of the source code” (4,m,Comp). Moreover, transparency is defined as a social effort and collaborative outcome involving government institutions such as health authorities, hospitals and vaccination centers as well as technology companies like Apple and Google and critical organizations like the European “Chaos Computer Club” (6,m,Leg). Transparency is considered successful when users are provided with information about “What kind of data is being collected? Where is it stored? How is it stored? How often is it deleted? And at the end of the day, what can you actually read about me?” (9,f,Soc) These inquiries also touch upon underlying concerns, elucidated in our research as anxiety regarding “being monitored as transparent citizens” (6,m,Leg) or worries “about the state having excessive information about them [the citizens].” (6,m,Leg) In this connection, the importance of transparency is discussed against the backdrop of “massive encroachments on fundamental rights” (14,f,Eth) such as curfews, distancing rules, and bans on assembly.

Given that mobile technologies are ubiquitous and interwoven with all different kinds of aspects of our lives, I think that there are grounds for the worry that this gives the one who controls these processes a tremendous amount of power. (3,m,Eth)

### Informed Consent

In contrast to this, transparency is considered a fundamental requirement by most interviewees for informed “self-determination” (11,m,Leg), which is seen as crucial for understanding the functioning of DCT, providing informed consent for their use and fostering “empowerment” (10,f,Med) by “enabling individuals to protect themselves if personal information is accessed that they believe is private.” (5,m,Eth) Moreover, almost all participants of our study emphasized that “in democratic, liberal societies … things that come via coercion, so to speak, and come via prohibitions” (9,f,Soc), do not work and can destroy people’s trust in “politics and society”. (9,f,Soc) In this context, transparency is considered a fundamental value to engage in an informed discourse where citizens need to be “convinced” (9,f,Soc) by information, “but not in the sense of persuading, but in the sense of: ‘Let me … tell you the advantages, I’ll tell you the disadvantages and now please decide whether this is’, so to speak, justifiable and applicable for you.” (9,f,Soc)

I believe that … in the long term, it is more effective to rely on voluntary participation and then say: ‘I offer very valuable information, don’t I? I make everything transparent. I am also the one who manages this warning app, very, very actively, and I take great care of the users’. (15,f,Med)

The function of transparency is then seen in assuring users that there is no „unauthorized data transmission” (13,f,Med) and that they need not „worry about being monitored.” (6,m,Leg) In this manner, transparency serves as the foundation for building trust in a technology that is used in various areas of everyday life, where sensitive data is entrusted without “obligation” (1,m,Journ) but rather through “communication of self-disclosure” (1,m,Journ).

In our study, informed consent is also tied to various prerequisites. It necessitates active acquisition and comprehension of information and may be accompanied by misconceptions, such as those regarding the epidemiological effectiveness of DCT. Additionally, informed usage entails users downloading the app (“opt-in”), maintaining the app and Bluetooth function active and undertaking various steps like uploading and sharing a positive test result.

Self-determination is therefore very high. … On the other hand, there is the discussion that it is perhaps too high. That quite a few people have this, yea …, that perhaps more automatisms should be built in. (12,m,Comp)

The voluntaristic approach is also contrasted with those based on an “opt-out” (10,f,Med), “where it is stated: ‘We are now installing the app on all smartphones, and anyone who doesn’t want it has to uninstall it’, … similar to organ donations.” (10,f,Med) In light of this, an effective pandemic response is seen as a balance between self-determination and heteronomy.

I also believe that there is a set of fundamental rights that set the frame within one can act so that … excludes at least some policies. In this case, I think a mandatory digital contact tracing policy would be contradict basic privacy rights. And in that regard, I also …, well that entails autonomy …, I would say. But I don’t think that enabling choice is necessarily in all cases the way to go. So, I do believe that there may be directive public health policies that can be justified even though they limit choices. (3,m,Eth)

At the same time, a connection between self-determination and the welfare state principle is established in our interviews. On one hand, it is assumed that self-determined use should “not jeopardize individual freedom” (7,f,Soc). On the other hand, it should also protect the “common good” (7,f,Soc). This raises the question of the extent to which users can be empowered to support other users by sharing their data and thus behave “cooperatively” (4,m,Comp) via technologies and in solidarity with others in the face of the pandemic.

Well, I can decide at any point whether I want to take part or not. I can decide whether to share my data. But that’s also a problem of the whole pandemic. So, to what extent am I willing to …, willing to give up my personal data for the common good? (7,f,Soc)

At the same time, informed consent is associated with several limitations which in our study are described as a “gap between an ideal of self-controlled, autonomous choice and mobile health practice in general.” (3,m,Eth) Therefore, self-determined use does not necessary imply that users comprehend “everything that occurs in relation to data, in terms of data processing” (11,m,Leg). Rather, it requires “prior knowledge” (14,f,Eth) and “digital literacy” (7,f,Soc). In this regard, a key point for transparency is recognized in the traceability without a “strong technical background” (17,m,Comp) and explained with a general understanding of “where each type of data is collected” (17,m,Comp). Transparency then means that the user, despite their limited understanding, can comprehend how DCT-apps work to the extent that they also agree with the implicit processes associated with such technology.

So, it’s like …, I don’t do my own TÜV [vehicle inspection in Germany] nor do I somehow check food safety myself. And suddenly, I’m supposed to be able to understand highly complex IT systems and assess the consequences of my data release. I find that a very, very questionable point of view. (4,m,Comp)

### Data Sovereignty

Particular emphasis is also placed on the aspect of impartial control of DCT which is referred to as “democratic technology development” (4,m,Comp). In our study, its necessity is justified by the concern that users of DCT might “reveal too much about themselves” (7,f,Soc) and thus become “transparent patients” (7,f,Soc). This skepticism is explained by a general lack of transparency regarding how external service providers such as “address marketers like Axiom” (4,m,Comp) and tech companies like “Google and Apple” (10,f,Med) handle user data. Simultaneously, it is attributed to government measures such as the aforementioned “census” (6,m,Leg), “monthly scandals” (4,m,Comp) about “data leaks” (4,m,Comp) or a general”mistrust” (1,m,Journ) in the government.

To counteract this, our study participants also call for the establishment of an infrastructure for “data sovereignty” (2,m,Eth) which is described as “fundamental traceability … of how user data is managed” (9,f,Soc) and includes legal provisions such as privacy and data protection (11,m,Leg), technical solutions like decentralized storage models (2,m,Eth / 7,f,Soc) or the provision of traceable information (6,m,Leg). Specifically, it is demanded that various aspects of app development such as the data storage model or user anonymization be made accessible as “open-source software” (4,m,Comp / 6,m,Leg) on platforms like “GitHub” (4,m,Comp), thus enabling “civil society scrutiny” (2,m,Eth).

I believe it’s positive that … at the development stage, so to speak, that everything was made open source, that A, the codes are accessible for everyone. Of course, that also increases transparency. Then, B, that they actually brought in outside voices in the development process right from the start. For example, the Chaos Computer Club, who looked at the thing and, it [the Corona-Warn-App] was initially planned as a centralized solution, and then, at the instigation of the Chaos Computer Club and data protectionists, they switched to a decentralized solution. (6,m,Leg)

There is a consensus among the study participants that an effective pandemic response depends on both the quality and quantity of data. Particularly, it is related to the acceptance of the app as “as little data as possible leads to greater acceptance and trust” (1,m,Journ). However, it is also noted that greater acceptance does not necessarily equate to greater effectiveness in epidemiological terms. Against this backdrop, a well-functioning approach to help combat the spread of the pandemic is recognized in establishing a smart balance between data invasiveness and epidemiological benefit. In this regard, particular attention is drawn to the relevance of GPS data which is not available in the case of the German DCT app. Epidemiologically, this data plays an important role as GPS data makes it possible to “identify local clusters more quickly” (1,m,Journ).

Yes, of course it would make it a lot easier to have GPS data because, … when I get a message saying: ‘You had a risk contact’ and I don’t even know where it was, so? Was it somehow on a trip to the Brandenburg Forest? Or was it when I was shopping in the supermarket around the corner? (15,f,Med)

### Digital Literacy

Transparency also requires not only access to information but an understanding of the information made transparent. In our study, this is associated with “digital literacy” (7,f,Soc) which is explained as a combination of “health literacy” (7,f,Soc) and “media literacy” (7,f,Soc). In this respect, various information generated by DCT such as the risk calculation must not only be technically prepared and processed but also understood by the users. To ensure this, cooperation between users, technology and experts is emphasized.

So, people call [a doctor] and say: ‘My warning app is red, what should I do?’ Then, of course, we are able to help them quite quickly or faster than if we had to wait for the result, the lab result … and actually give people a swab appointment quickly. (13,f,Med)

In addition, two further aspects are emphasized.

1. *Personal Requirements:* To comprehend the disclosed information it is essential to have the know-how to access the information. It is therefore important that “the population is aware … that transparency essentially exists” (15,f,Med) and that users can access this information and gain knowledge. The latter is also described in our study as a “burden of self-determination” (11,m,Leg): “So you could also say that consent requires me to inform myself in a world where we all experience a lot of stress.” (11,m,Leg)
2. *Technical Requirements:* Additionally, the technology itself serves as a mediator of skills as users rely on comprehending technical instructions and performing tasks like tests and vaccinations based on “quick information” (8,f,Med) without the need to “read through all the background information” (8,f,Med). Although the information may be communicated transparently, the “user guidance within the app itself can confuse many people” (1,m,Journ). This is exemplified by a lack of other languages included in DCT apps or when those fail to “communicate in a comprehensible language” (18,m,Soc). It is also evident when abstract formulations such as high or low-risk warnings are not understood by users (8,f,Med / 4,m,Comp) or physical symptoms appear even though no warnings were displayed on the smartphones.

There is one big problem. There is an intermediate stage between ‘no risk’ and ‘high risk’. No risk means: ‘Go out, do sport, feel free.’ And high risk means: ‘Go self-quarantine’, so to speak. But there is an intermediate stage and I think it is called ‘low risk’. And the forums and Twitter and feedback stories are full of users who simply don’t know what that means. (4,m,Comp)

This point also pertains to a general phenomenon of how health technologies communicate about diseases. In view of the incalculable risks and consequences of a COVID-19 infection, receiving infection reports “via the smartphone can be shocking” (14). At the same time, they refer to “different horizons of understanding” (5) as they do not provide an opportunity to counsel a “diagnosing doctor” (15) and thus verify the infection.

There are various levels of understanding. One person who is technically adept might say: ‘Okay, that's a certain score, I don’t need to worry.’ Another person looks at it and is immediately terrified, starts trembling, thinking: ‘Oh, I’m about to die, what's happening?’ and becomes completely panicked, locks inside his house and refuses to go out. … This is a communication problem, as I said. Think of Dr. House. He only tells the truth: ‘You have cancer and have an 85% chance of dying in the next 6 months’ (5).

### Social Asymmetries

Furthermore, the interviewees reflected on transparency at various levels concerning its social asymmetries citing examples such as the dependence of governments implementing DCT on tech companies, the availability of smartphones or the citizens’ knowledge of using DCT. On one hand, “the whole development on different platforms [is] something that the state itself cannot afford – in this respect, we need these forms that actually carry out the technical implementation.” (17,m,Comp) On the other hand, the state’s reliance on tech companies complicates the ability to forecast how they will manage their expenses and, if needed, handle personal data in the future.

But there is one area of the app that is not discussed and that is not transparent … and that is: What will Google and Apple actually do with my data? (6,m,Leg)

Do I trust a democratic state more, which is at least in some way tied back and there is accountability and all that sort of things, or do I trust companies that I can’t control at all? (9,f,Soc)

The participants also address two other interlinked factors of social inequality. In this regard, the issue of widespread social exclusion from the technological fight against the pandemic is being discussed. This includes the exclusion of vulnerable groups of people who “do not own a smartphone at all” (8,f,Med), “lack a stable internet connection” (14,f,Eth), “reside in rural areas” (7,f,Soc), “experience inadequate network coverage in terms of infrastructure” (7,f,Soc) or “no longer receive updates [on their smartphones]” (8,f,Med).

When we talk about justice: Who has the newer phones? Who has the latest smartphones where everything runs smoothly? It’s just not fairly distributed … Even an iPhone from 2012 is not inexpensive. And then the competence is not evenly distributed either. So, how can I assess how my data is being used? How it’s stored? What is being transmitted? You have to understand that first. (7,f,Soc)

In this regard, the situations of people who are “homeless” (14,f,Eth) and due to their social circumstances “at very high risk” (14,f,Eth) and “simply not in good enough health to counteract an infection very well” (14,f,Eth) are also being highlighted. The stated vulnerable groups of people also include “elderly people who are not familiar with modern technology, residing in nursing homes or facilities for the disabled” (13,f,Med) as well as “children” (8,f,Med) who have contact with other children and adults and often do not have smartphones. This also applies to refugees and ‘LGBTQI+ people, who are already more affected by digital violence anyway, and are therefore more skeptical about sharing their data’ (9,f,Soc). The social and technological factors are thus intertwined to the extent that vulnerable groups of people who need to be protected are among those who can be excluded from the technological fight against the pandemic due to their age, income, gender, language, economic status or cultural backgrounds.

That means I need a smartphone and I need to be able to use one. And that is not something that can be taken for granted among the over-70s. And secondly, it also has to be a modern smartphone. This implies that some individuals with limited economic power may encounter difficulties. In other words, things come together. I have to have enough financial means and understanding of technology to really be able to utilize it. (12,m,Comp)

Finally, our study participants reflect on various proposed solutions and improvements for implementing DCT across the population. The proposals include concrete suggestions such as the introduction of “Corona App Dongles” (4,m,Comp) which people can carry with them in the form of a “small device … on which only this app runs” (4,m,Comp), “Bluetooth transmitters that you have in your pocket for single-purpose use only, so that you can participate in the system” (11,m,Leg) as well as the proposal to distribute digital wristbands whose sole purpose is to run this contact tracing.” (1,m,Journ).

## Discussion

DCT has been viewed by many societies as a crucial technology to mitigate a severe health crisis. Its effectiveness is multifaceted: it can engage people in a continuous information exchange by using a widespread device such as the smartphone, create a network among millions of people based on non-physical contact aimed at detecting and predicting infections and reduce the burden on the healthcare system. Given the scenario of societal vulnerability, high standards of transparency in the handling of user data are required so that people trust an unfamiliar health technology and believe that sharing personal data will protect their lives and the lives of others. Our findings underscore that the new technological measure for pandemic preparedness are recognized as a promising field but at the same time are also critically questioned. In the case of voluntary use, users of a new health technology need not only to be convinced of its benefits but also to be assured that various requirements, such as privacy standards (e.g., how data is generated, stored, and processed), will be met. In a broader perspective, these aspects are also linked to a general reliability of the governments, companies and researchers that stand behind the development of a health technology. In this regard, transparency plays a key role as part of a technical requirement aimed at securing control on the side of users, convincing them to use a new technology and providing them with an “informed voice” [10, 43]. Summarizing our results we identified four key areas. In order to gain the trust in new technological measure under the condition of voluntary usage it is important to:

1. **Communicate complex processes** such as data collection and dissemination openly and comprehensibly and also provide information on where users can find relevant information such as comprehensible explanations. In this regard, our findings align with other scientific results emphasizing the importance of implementing strategies aimed at reducing uncertainty in health technology [33, 39], particularly within the context of DCT [1, 42]. As outlined by Oldeweme et al. [33], transparency can then be viewed as a strategy to alleviate uncertainty and foster acceptance among individuals by empowering them through processes of understanding and informed decision-making. At the same time, our data indicates that communication goes far beyond the question of how to generate acceptance and avoid insecurity. From a more social science and ethical perspective, it is part of a societal and individual necessity based on the principle of a “democratic technology development” (4,m,Comp) and a fundamental desire to avoid becoming “transparent patients” (7,f,Soc). Especially during the pandemic, the need for open and comprehensible communication can also be seen as a factor of avoiding “mistrust” (1,m,Journ) by realizing a “civil society scrutiny” (2,m,Eth) and by establishing users as sovereign actors.
2. **Protect private data** through storage models that secure against third-party access and future risks. For example, Abeler et al. [1] and Dar et al. [7] highlight concerns among individuals using DCT about the potential misuse of personal data to enforce quarantine measures or restrict access to public areas. These studies underscore the concern that individuals using DCT may experience social pressure or fear exclusion based on their decision to use or not use such technology [8]. At the same time, our findings also make clear that the trust in handling of one’s data is primarily dependent on how the central actors behind the development of a technology (a.o. tech companies like Google and Apple and governments) manage a technology. “Do I trust a democratic state more, which is at least in some way tied back, and there is accountability and all that sort of things, or do I trust companies that I can’t control at all?” (9,f,Soc) Additionally, the study repeatedly raises the question of the meaningfulness of the existing data generation: While one social group which has access to knowledge and is equipped with the appropriate technological devices is confronted with privacy requirements, vulnerable groups in the pandemic, such as the “homeless” (14,f,Eth), elderly or children, are often excluded from such technological measures [13, 21, 28]. Similar arguments are made by Ranisch et al. [16], who state that in order to justify the use of DCT, there must be proportionality, consisting of health necessity, technological effectiveness and minimization of ethical risks. This can be achieved, in addition to transparent communication, primarily through reliability, such as technological procedures for correct calculation of positive results and avoidance of false results, which, in worst case scenarios, may lead to “higher morbidity and mortality and greater economic damage” [36].
3. **Include critical perspectives** from users and organizations who were not directly involved in the development of a public technology and consider their feedback in further updates. In critical research, the importance of “data minimalism” as a strategy against widespread “data expansionism” [37] is emphasized in this context. For example, Richterich illustrates how hacker associations worldwide such as the Chaos Computer Club became involved in the early phase of the COVID-19 pandemic and published recommendations for transparent communication of data generation as well as for “low(er)-tech options”. Their suggestions were particularly aimed at potential data invasiveness which could spread widely under the pretext of epidemiological urgency and, in doing so, undermine fundamental aspects of autonomy and privacy [3]. In addition, in our study it is pointed out that transparency constitutes an ongoing collaborative practice in which various institutions such as hospitals, vaccination centers, technology companies and critical organizations work together. The critical instances thereby have the function of impartially highlighting processes and shortcomings, thereby also ensuring the trust of individuals not technically inclined.
4. **Create a user-friendly design** and interface that does not overwhelm users but instead motivates them to adopt and interact with a health technology [42]. Research on the role of User Experience Design (UX Design) indicate that perceived usefulness, trustworthiness and privacy protection are perceived as the most important aspects of using DCT [34]. For example, Oyibo and Morita [34] demonstrate that people are more likely to engage with DCT when they trust that it truly helps them and that their data is handled confidentially. Factors such as enjoyment in using these technologies are considered less important. Our study also points out that effective pandemic control requires the use of technology that can be understood and used even without a “strong technical background” (17, m, Comp). In a pandemic situation, the goal of the design should be to provide “quick information” (8, f, Med) in a comprehensible and confidential manner without the need to “read through all the background information” (8, f, Med).

## Data Availability

The interviews and transcribed qualitative data are stored on an external storage medium at the Ruhr-University Bochum.

## Footnotes

1 The coding represents the status as of April, 2024. The empirical data is being further analyzed within the ELISA-project.
